# Rifampicin Resistance Pattern of Mycobacterium tuberculosis Infection in Tertiary Care Hospital Settings

**DOI:** 10.7759/cureus.55755

**Published:** 2024-03-07

**Authors:** Avantika Ranganathan, Durai Singh Carmelin, Raman Muthusamy

**Affiliations:** 1 Center for Global Health Research, Saveetha Medical College and Hospitals, Saveetha Institute of Medical and Technical Sciences, Saveetha University, Chennai, IND

**Keywords:** multi-drug-resistance organism, tertiary care hospital, rifampicin, antimicrobial resistance, mycobacterium tuberculosis

## Abstract

Introduction

*Mycobacterium tuberculosis *(MTB), the causative agent of tuberculosis (TB), continues to pose a significant global health threat, with increasing concerns about antimicrobial resistance (AMR). This study aims to elucidate the AMR patterns of MTB infections in tertiary care hospital settings.

Materials and methods

A retrospective analysis was conducted on 138 clinical samples collected from patients attending the outpatient ward with clinically suspected MTB infections from November 2022 to April 2023 in a tertiary care hospital, Saveetha Medical College and Hospital. The study focused on the sample isolates collected from various clinical specimens, such as sputum, pus, synovial fluid, wound swabs, and other forms of samples from the patients. The samples were processed and analyzed with routine microbiological confirmation tests using standard laboratory methods such as staining and culture. Further, the samples were subjected to a GeneXpert MTB/RIF assay to assess the resistance to Rifampicin (RIF). The results were interpreted, analyzed using standard statistical methods, and presented.

Results

The findings revealed marked resistance of the clinical isolate MTB to TIF, with positive and negative results through various peak levels shown by GeneXpert. Out of the 138 samples screened by GeneXpert for resistance, 14 samples were found to be positive (10.14%). Resistance to the first-line drug, namely RIF, was observed in the study, raising concerns about the effectiveness of standard tuberculosis treatment regimens followed in the country.

Conclusion

This study implies the urgency of monitoring and addressing AMR in MTB infections in tertiary care hospital settings. The emergence of resistance to even the first-line drugs necessitates continuous surveillance, the implementation of appropriate diagnostic strategies, and the development of effective treatment protocols. A comprehensive understanding of the AMR landscape in tuberculosis is crucial for optimizing therapeutic interventions, preventing the spread of drug-resistant strains, and ultimately curbing the global burden of tuberculosis.

## Introduction

Tuberculosis is an infectious disease caused by the bacterium *Mycobacterium tuberculosis* (MTB). It is highly contagious and primarily affects the lungs, resulting in the characteristic symptoms of pulmonary tuberculosis. However, it can also affect other organs and tissues, such as the lymph nodes, brain, kidneys, and spine, which is known as extrapulmonary tuberculosis [1]. Based on the global report by the World Health Organization (WHO), approximately 5% of the world population shows immunologic evidence of past infection with MTB* *through surveillance testing. In the year 2020, an estimated 10 million individuals were diagnosed with active tuberculosis (ATB). Especially, multidrug-resistant tuberculosis (MDR-TB) has emerged as a significant global public health issue, affecting approximately 450,000 individuals worldwide and demonstrating a mortality rate surpassing that of cancer [2]. Thus, tuberculosis is recognized as one of the most fatal infections worldwide [3].

Primarily the treatment begins with the appropriate antimicrobial drugs. The utilization of antimicrobial drugs in modern medicine has aided in the treatment of infectious diseases caused by bacteria, fungi, parasites, and viruses. However, the repeated use of the drugs in various settings, including human, animal, and environmental, has led to the emergence of antimicrobial resistance (AMR) in different types of microorganisms. Consequently, AMR has become a significant concern for public and environmental health over the last two decades, posing a substantial threat [4]. The timely identification of drug resistance is of utmost importance to prevent the spread of drug-resistant tuberculosis and reduce mortality rates. However, in low- and middle-income countries, the establishment of effective tuberculosis resistance diagnostic programs that utilize molecular tools presents a significant challenge [5].

Prior to the treatment, it is essential to detect the infection in the initial stage and then commence with the medication. As mentioned earlier, a significant portion of the population shows indications of past or ongoing infection with MTB. To diagnose either latent tuberculosis infection (LTBI) or ATB, various assays are available depending on the clinical status. Immunological techniques currently used for diagnosing LTBI or ATB include the Mantoux Tuberculin Skin Test (TST) and interferon-gamma (IFN-γ) release assays. These methods help healthcare professionals, especially in tertiary care hospitals to assess the presence of tuberculosis infection and to determine the appropriate treatment strategy [6]. Nevertheless, the use of the Bacillus Calmette-Guerin (BCG) vaccine in children has been ineffective in protecting the children against the primary complex of tuberculosis and tuberculosis infection in adults [7,8].

The current standard treatment for drug-susceptible tuberculosis involves a combination of antibiotics, including rifampicin (RIF), isoniazid (INH), pyrazinamide (PZA), and ethambutol (EMB) for the initial two months, which is considered the first line of medications. This is followed by a continuation phase of four months with RIF and INH [9]. Indiscriminate inappropriate and under-dosing of the above combination of drugs with a frequent pause in the medication period leads to drug resistance of MTB resistant initially to RIF; while such discontinuity in medication with a combination of drugs for a longer duration leads to extensively drug-resistant MTB [10].

Traditional diagnostic assays that are labor-intensive and time-consuming are being substituted with advanced molecular tests in the field of diagnostics. One such test is the GeneXpert - a nucleic acid-based test, which not only detects the presence of MTB in the sputum, as well as other body fluids of patients with ATB but also identifies gene that is resistant to RIF, a crucial first-line drug for ATB treatment. Consequently, in locations where this test is accessible, patients who are suspected of having ATB can receive a prompt molecular diagnosis and be prescribed a more efficient initial treatment, especially in cases of drug-resistant tuberculosis [11,12]. This study undergoes by identification of the positive MTB which is resistant to the first-line drug RIF and provides a treatment plan and control measures that need to be taken with the help of the data collected.

## Materials and methods

Materials and chemicals

Blood agar, Lowenstein Jensen (LJ) medium, RIF, and biochemical reagents were purchased from Hi-Media (Mumbai, India). A standard MTB strain was obtained from the Department of Microbiology, Saveetha Medical College. GeneXpert MTB/RIF was performed at the Saveetha Medical College and Hospital Laboratory Complex.

Sample collection

Clinical specimens such as sputum, pus, synovial fluid, wound swabs, and other forms of samples were collected from the patients suspected of tuberculosis infection attending the tuberculosis and chest department wards of the tertiary health care setting of Saveetha Medical College and Hospitals (SMCH). Following all the stipulated ethical guidelines, ethical clearance was obtained from the Institutional Review Board and Ethics Committee (IRB No. 112101168).

Methodology

A retrospective study was performed on 138 samples of patients undergoing clinical diagnosis at Saveetha Medical College and Hospital from November 2022 to April 2023. The study received approval from the institutional review board and adhered to ethical guidelines for research involving human subjects, as outlined in the Helsinki Declaration [13] and subsequent amendments or similar ethical standards. Prior to participation, informed consent was obtained from all individuals involved in the study. Clinical specimens, such as pleural fluid, sputum, bronchoalveolar lavage, pus, fine-needle aspiration cytology (FNAC), and urine samples were collected if the patient was suspected to have a tuberculosis infection. Medical records and relevant imaging were reviewed for intervention, and microbiological testing was performed.

Sample processing

**Figure 1:**
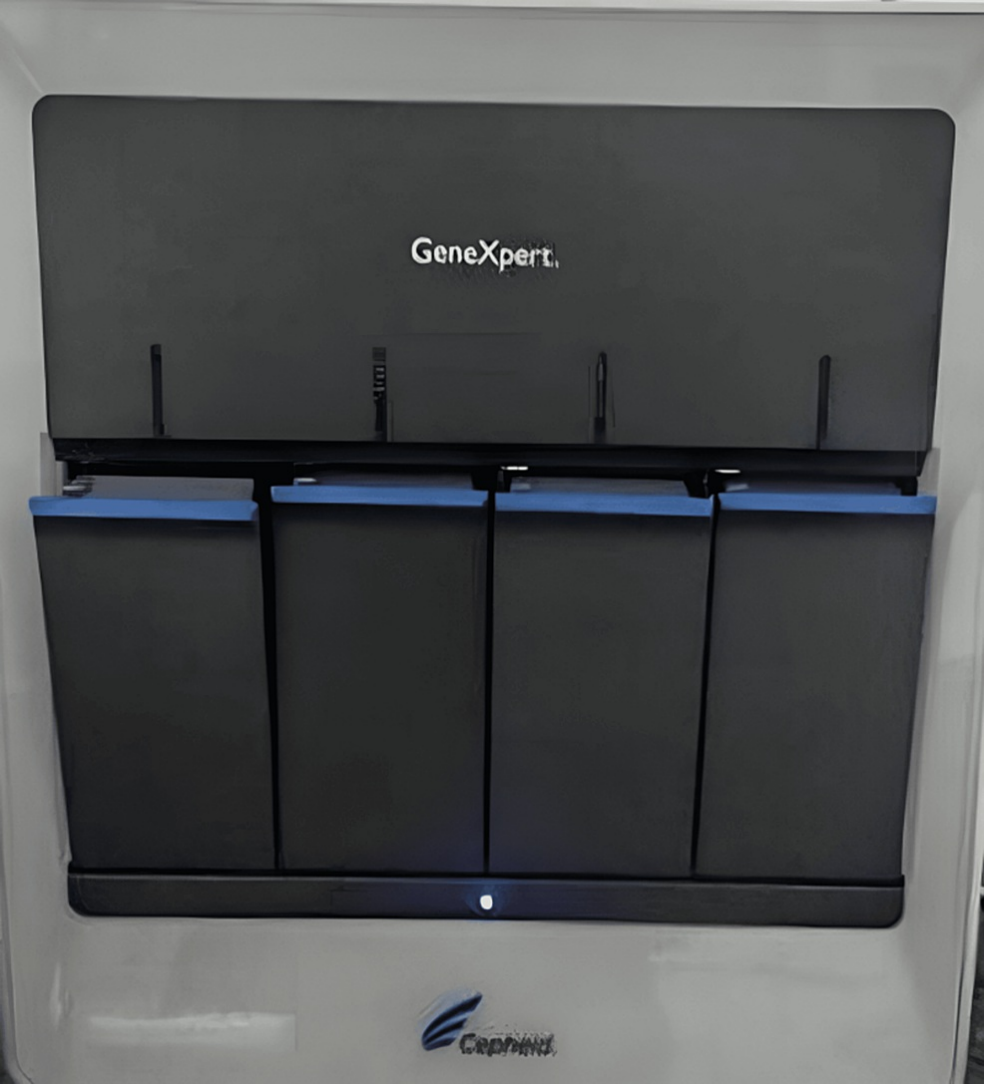
GeneXpert Diagnostic device to detect Mycobacterium tuberculosis Diagnostic device GeneXpert was performed at Saveetha Medical College and Hospitals

The obtained samples were analyzed using the GeneXpert MTB/RIF assay (Figure 1). Samples were transferred into 15mL falcon tubes, and buffer was added in a 2:1 ratio. The tubes were then agitated manually twice during the 15 minutes of incubation at the ambient temperature of 37 °C. Then 2mL of the material was transferred into the cartridges with the disposable pipettes available in the kits, and then they were loaded into the machine. Samples were filtered and washed automatically, and the filtered sample underwent ultrasonic lysis, which caused the organism to release the DNA, which was further mixed with the dry PCR reagents. Semi-nested real-time amplification and detection take place in the integrated reaction tube. The results were analyzed and updated on the computer after two hours of processing.

Drug-sensitivity testing

Drug sensitivity testing was conducted using the proportion method on LJ medium. The time required to obtain updated results for the smear was approximately one to two hours, while the GeneXpert MTB/RIF assay took around three to four hours. Results from the *Mycobacterium *growth indicator tube were obtained after four to six weeks, and culture along with the drug sensitivity testing yielded results after eight to 10 weeks.

Compliance with ethical standards

The institute review board for ethical concerns approved the study. This study was performed in accordance with the Declaration of Helsinki and its further amendments to comparable standards [13].

## Results

**Figure 2:**
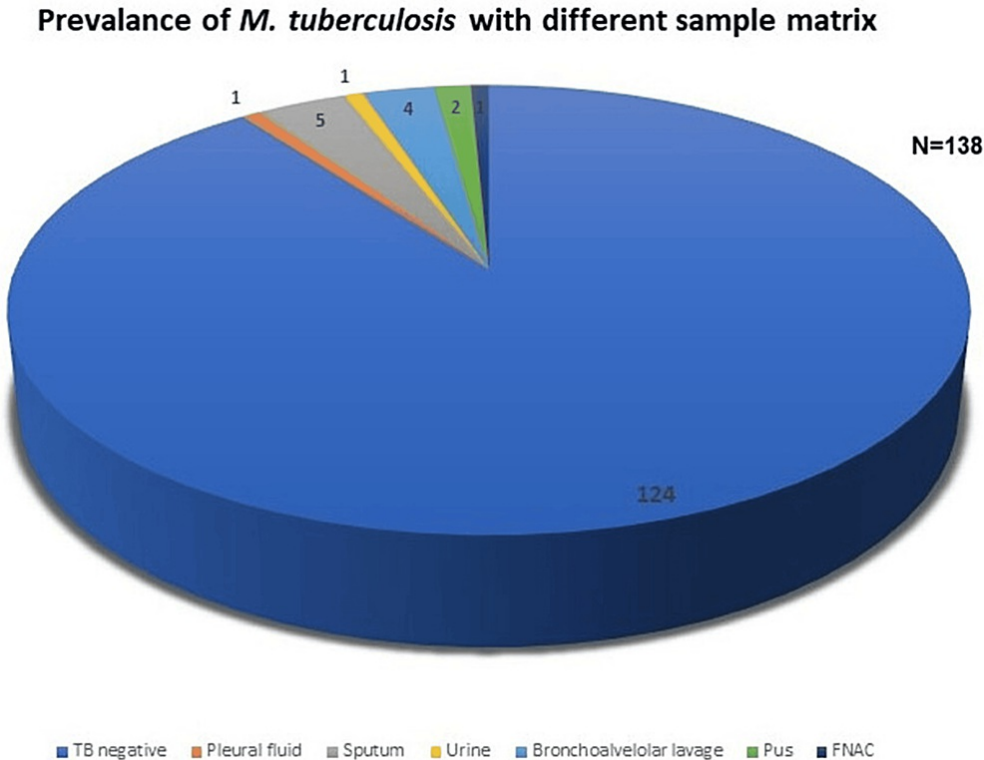
Positive and negative results obtained from different sample matrix The figure denotes the total number of samples tested and number of positive and negative results obtained from various types of samples collected and “N” denotes total number of samples collected FNAC - Fine-Needle Aspiration Cytology

Out of 138 samples evaluated (Table 1), pleural fluid one (0.72%), sputum five (3.62%), bronchoalveolar lavage one (0.72%), pus four (2.89%), FNAC two (1.44%), urine one (0.72%) and tuberculosis negative samples were found to be 124 (Figure 2, Table 2).

**Table 1:** Total number of samples collected, negative, and positive results

Sl.No.	Types of sample collected	Negative for MTB	Positive for Rifampicin
	Sputum	Negative	Negative
	Urine	Negative	Negative
	Pleural fluid	-	Positive
	Sputum	Negative	Negative
	Bronchoalveolar lavage	Negative	Negative
	Pleural fluid	Negative	Negative
	Urine	Negative	Negative
	Bronchoalveolar lavage	Negative	Negative
	Sputum	-	Positive
	Pus	Negative	Negative
	Urine	Negative	Negative
	Bronchoalveolar lavage	Negative	Negative
	Sputum	-	Positive
	Pus	Negative	Negative
	Sputum	Negative	Negative
	FNAC	Negative	Negative
	Pleural fluid	Negative	Negative
	Urine	Negative	Negative
	Bronchoalveolar lavage	Negative	Negative
	Urine	Negative	Negative
	Bronchoalveolar lavage	Negative	Negative
	Pleural fluid	Negative	Negative
	Sputum	-	Positive
	FNAC	Negative	Negative
	Pleural fluid	Negative	Negative
	Sputum	Negative	Negative
	Pus	Negative	Negative
	Sputum	Negative	Negative
	FNAC	Negative	Negative
	Bronchoalveolar lavage	Negative	Negative
	Sputum	Negative	Negative
	Pleural fluid	Negative	Negative
	FNAC	Negative	Negative
	Urine	Negative	Negative
	Bronchoalveolar lavage	Negative	Negative
	Pleural fluid	Negative	Negative
	Sputum	Negative	Negative
	FNAC	Negative	Negative
	Bronchoalveolar lavage	Negative	Negative
	Sputum	Negative	Negative
	Pleural fluid	Negative	Negative
	Pus	Negative	Negative
	Urine	-	Positive
	Bronchoalveolar lavage	Negative	Negative
	Sputum	Negative	Negative
	Pus	Negative	Negative
	Bronchoalveolar lavage	Negative	Negative
	Sputum	-	Positive
	Urine	Negative	Negative
	FNAC	Negative	Negative
	Sputum	Negative	Negative
	Bronchoalveolar lavage	Negative	Negative
	Urine	Negative	Negative
	Pus	Negative	Negative
	Bronchoalveolar lavage	Negative	Negative
	Sputum	Negative	Negative
	Pus	Negative	Negative
	Bronchoalveolar lavage	Negative	Negative
	Sputum	Negative	Negative
	FNAC	-	Positive
	Urine	Negative	Negative
	Pus	Negative	Negative
	Sputum	Negative	Negative
	Bronchoalveolar lavage	Negative	Negative
	Pleural fluid	Negative	Negative
	FNAC	Negative	Negative
	Sputum	Negative	Negative
	Bronchoalveolar lavage	Negative	Negative
	Urine	Negative	Negative
	Pleural fluid	Negative	Negative
	Sputum	Negative	Negative
	Bronchoalveolar lavage	Negative	Negative
	Sputum	Negative	Negative
	Pus	Negative	Negative
	Urine	Negative	Negative
	Sputum	Negative	Negative
	Pleural fluid	Negative	Negative
	FNAC	Negative	Negative
	Urine	Negative	Negative
	Bronchoalveolar lavage	Negative	Negative
	FNAC	Negative	Negative
	Sputum	Negative	Negative
	Pleural fluid	Negative	Negative
	FNAC	Negative	Negative
	Sputum	Negative	Negative
	Bronchoalveolar lavage	Negative	Negative
	Pleural fluid	Negative	Negative
	Sputum	Negative	Negative
	Pus	Negative	Negative
	Urine	Negative	Negative
	FNAC	Negative	Negative
	Sputum	-	Positive
	Pleural fluid	Negative	Negative
	Pleural fluid	Negative	Negative
	Sputum	Negative	Negative
	Pus	Negative	Negative
	Pleural fluid	Negative	Negative
	Bronchoalveolar lavage	Negative	Negative
	Sputum	Negative	Negative
	Pus	Negative	Negative
	Bronchoalveolar lavage	Negative	Negative
	Sputum	Negative	Negative
	Pleural fluid	Negative	Negative
	FNAC	Negative	Negative
	Pus	-	Positive
	Sputum	Negative	Negative
	Pus	-	Positive
	Sputum	Negative	Negative
	Pleural fluid	Negative	Negative
	Bronchoalveolar lavage	-	Positive
	Sputum	Negative	Negative
	Pleural fluid	Negative	Negative
	Pleural fluid	Negative	Negative
	Pus	-	Positive
	Sputum	Negative	Negative
	Sputum	Negative	Negative
	Pleural fluid	Negative	Negative
	Pus	-	Positive
	Sputum	Negative	Negative
	FNAC	Negative	Negative
	Sputum	Negative	Negative
	Pleural fluid	Negative	Negative
	FNAC	Negative	Negative
	Sputum	Negative	Negative
	Urine	Negative	Negative
	Sputum	Negative	Negative
	Pleural fluid	Negative	Negative
	Urine	Negative	Negative
	Sputum	Negative	Negative
	Bronchoalveolar lavage	Negative	Negative
	FNAC	-	Positive
	Urine	Negative	Negative
	Sputum	Negative	Negative
	Urine	Negative	Negative
	Pleural fluid	Negative	Negative
	Urine	Negative	Negative
	Sputum	Negative	Negative
	Sputum	Negative	Negative

**Table 2:** Positive and negative results confirmed from various types of samples collected (N=138) FNAC - Fine-Needle Aspiration Cytology

Types of samples tested	Number of samples	Positive for rifampicin resistance	Positive in percent (%)
Pleural fluid	24	1	0.72
Sputum	40	5	3.62
Bronchoalveolar lavage	20	1	0.72
Pus	12	4	2.89
FNAC	13	2	1.44
Urine	15	1	0.72

**Figure 3:**
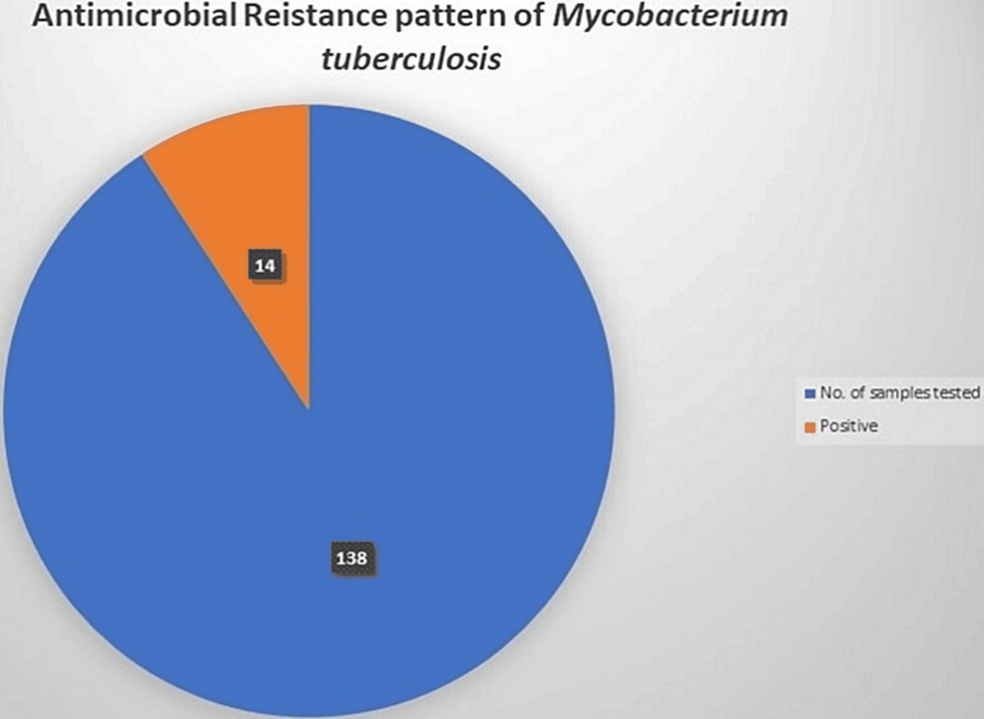
Antimicrobial resistance pattern of M. tuberculosis The figure denotes the number of samples tested or antimicrobial resistance of M. tuberculosis along with the number of positive cases and represented in percent (%).

Totally, 14 samples showed positive results (10.14%) (Table 3). The rifampicin resistance pattern of MTB was detected by GeneXpert. The presence of MTB, the DNA molecule of the bacteria, and the percentage of samples which is responsible for the resistance to the first-line drug Rifampicin were successfully detected (Figure 3).

**Table 3:** Susceptibility of M. tuberculosis against the antibiotic drug Rifampicin (N=138)

Number of samples collected	Positive results	Percentage
138	14	10.14

## Discussion

This study was carried out with 138 patient samples, out of which 14 (10.14%) samples showed positive results for RIF resistance. Other similar studies showed 86 (68.8%) positive results out of 125 samples of tissue biopsies [13]. Additionally, another relevant study reported, that out of 170 patients diagnosed with tuberculosis, 11 (6.47%) were found to be RIF resistant [14]. Therefore, detecting MTB and drug-resistant strains at an earlier stage is crucial for the prompt initiation of anti-tuberculosis treatment, ensuring better control of the infection [15]. Initially, studies relied on conventional methods based on solid culture media, which resulted in longer turnaround times for obtaining results [16]. Later, the diagnosis method stepped toward nucleic acid amplification (NAA)-based techniques. There has been a notable improvement in the performance of NAA tests. These tests, developed and conducted within a specific laboratory, have shown advancements in their accuracy and reliability [17]. Our study was carried out using a recent advancement technique, the GeneXpert MTB/RIF assay, which is a rapid and automated molecular test that can be conveniently performed near the point of care, allowing for early diagnosis of tuberculosis [18]. The main benefit of the study is that it is conducted within an automated cartridge, which significantly reduces the risk of contamination. This approach enhances the overall biosafety measures associated with handling such an infectious agent [19]. One of the other key advantages is that it provides simultaneous information on the potential drug resistance to RIF, a commonly used anti-tuberculosis medication [20]. This feature enables healthcare providers to quickly identify drug-resistant strains of tuberculosis and make informed decisions regarding appropriate treatment options [14].

Previous studies reported that the main reason for the RIF resistance against MTB due to mutation caused by specific codons (507-533) of the rpoB gene, which encodes the RNA polymerase beta subunit [21]. This specific region, known as the RIF resistance-determining region (RRDR), is the main target for modern molecular-based assays used to detect RIF resistance. Mutations in this region account for approximately 96% of RIF resistance cases [22]. Additional research studies have indicated that a portion of RIF resistance does not show any alterations in the rpoB gene. This suggests the presence of alternative mechanisms contributing to RIF resistance [23]. Additional studies report that changes in the amino acids surrounding the binding pocket for RIF in the active site of RNA polymerase lead to alterations in the active site, resulting in resistance to RIF [24]. As an alternative way of treatment for patients with RIF-resistant MTB*,* a regimen with at least five effective anti-tuberculosis drugs during the intensive phase is recommended, which includes PZA and four core second-line anti-tuberculosis drugs Each one is from Group A and Group B, and two drugs are from Group C [25]. It is clearly understood that it is necessary to closely monitor the tertiary care hospital, focus on the RIF-resistant MTBstrains by earlier identification using advanced techniques, and initiate alternative treatment for the emergence of the drug-resistant strains.

With increased availability and access to many classes of antibiotics from the access, watch, and reserve groups, the probability of drug resistance is relatively high in any given tertiary healthcare hospital setting. However, a larger number of samples need to be screened and evaluated for sensitivity and resistance status to arrive at the appropriate resistance status. Moreover, the samples for a few of the cases were not available with the discontinuation of the patients receiving the antibiotic medication among the medical records available. Hence, further studies with a larger sampling size would shed more light on the precise status of antibiotic resistance in patients suffering from MTB*.*

## Conclusions

In conclusion, the AMR pattern of MTB infection in tertiary care hospital settings is a significant concern. The emergence of drug-resistant strains such as MDR-TB and extensively drug-resistant tuberculosis poses a serious threat to public health. Understanding the molecular mechanisms of drug-resistant tuberculosis is crucial in mitigating the spread of drug-resistant strains, shortening treatment duration, minimizing adverse drug effects, and enhancing treatment outcomes for patients. This is especially important as the threat of tuberculosis to public health has intensified due to the emergence of drug-resistant tuberculosis, including MDR-TB and extensively drug-resistant tuberculosis, despite a global decline in infection rates. It is imperative for healthcare providers in tertiary care hospitals to closely monitor and address the AMR patterns of MTB to ensure appropriate and timely interventions. Continued research and collaboration are essential to combating these global health challenges.

## References

[1] Pai M, Behr MA, Dowdy D (2016). Tuberculosis. Nat Rev Dis Primers.

[2] Mohamed MA, Ali OA, Osman AM (2023). Assessment of drug-susceptible and multidrug-resistant tuberculosis (MDR-TB) in the Central Region of Somalia: a 3-year retrospective study. PLOS Glob Public Health.

[3] Bourzac K (2014). Infectious disease: beating the big three. Nature.

[4] da Silva Dantas A (2022). Antimicrobial resistance. Mol Microbiol.

[5] Elion Assiana DO, Abdul JB, Linguissi LS (2021). Epidemiological profile of multidrug-resistant and extensively drug-resistant Mycobacterium tuberculosis among Congolese patients. Ann Clin Microbiol Antimicrob.

[6] Pai M, Behr M (2016). Latent Mycobacterium tuberculosis infection and interferon-gamma release assays. Microbiol Spectr.

[7] Fatima S, Kumari A, Das G, Dwivedi VP (2020). Tuberculosis vaccine: a journey from BCG to present. Life Sci.

[8] Dockrell HM, Butkeviciute E (2022). Can what have we learnt about BCG vaccination in the last 20 years help us to design a better tuberculosis vaccine?. Vaccine.

[9] Mushtaq F, Raza SM, Ahmad A, Aslam H, Adeel A, Saleem S, Ahmad I (2023). Antimicrobial drug resistant features of Mycobacterium tuberculosis associated with treatment failure. PLoS One.

[10] Ramachandran G, Swaminathan S (2015). Safety and tolerability profile of second-line anti-tuberculosis medications. Drug Saf.

[11] Peloquin CA, Davies GR (2021). The treatment of tuberculosis. Clin Pharmacol Ther.

[12] Quenard F, Fournier PE, Drancourt M, Brouqui P (2017). Role of second-line injectable antituberculosis drugs in the treatment of MDR/XDR tuberculosis. Int J Antimicrob Agents.

[13] Karthek V, Bhilare P, Hadgaonkar S, Kothari A, Shyam A, Sancheti P, Aiyer SN (2021). Gene Xpert/MTB RIF assay for spinal tuberculosis- sensitivity, specificity and clinical utility. J Clin Orthop Trauma.

[14] Patel J, Upadhyay M, Kundnani V, Merchant Z, Jain S, Kire N (2020). Diagnostic efficacy, sensitivity, and specificity of Xpert MTB/RIF assay for spinal tuberculosis and rifampicin resistance. Spine (Phila Pa 1976).

[15] Mokrousov I, Otten T, Vyshnevskiy B, Narvskaya O (2003). Allele-specific rpoB PCR assays for detection of rifampin-resistant Mycobacterium tuberculosis in sputum smears. Antimicrob Agents Chemother.

[16] Idigoras P, Beristain X, Iturzaeta A, Vicente D, Pérez-Trallero E (2000). Comparison of the automated nonradiometric Bactec MGIT 960 system with Löwenstein-Jensen, Coletsos, and Middlebrook 7H11 solid media for recovery of mycobacteria. Eur J Clin Microbiol Infect Dis.

[17] Noordhoek GT, Mulder S, Wallace P, van Loon AM (2004). Multicentre quality control study for detection of Mycobacterium tuberculosis in clinical samples by nucleic amplification methods. Clin Microbiol Infect.

[18] Sharma SK, Ryan H, Khaparde S (2017). Index-TB guidelines: guidelines on extrapulmonary tuberculosis for India. Indian J Med Res.

[19] Rachow A, Zumla A, Heinrich N (2011). Rapid and accurate detection of Mycobacterium tuberculosis in sputum samples by Cepheid Xpert MTB/RIF assay--a clinical validation study. PLoS One.

[20] Lawn SD, Zumla AI (2011). Tuberculosis. Lancet.

[21] Telenti A, Imboden P, Marchesi F, Schmidheini T, Bodmer T (1993). Direct, automated detection of rifampin-resistant Mycobacterium tuberculosis by polymerase chain reaction and single-strand conformation polymorphism analysis. Antimicrob Agents Chemother.

[22] Caws M, Duy PM, Tho DQ, Lan NT, Hoa DV, Farrar J (2006). Mutations prevalent among rifampin- and isoniazid-resistant Mycobacterium tuberculosis isolates from a hospital in Vietnam. J Clin Microbiol.

[23] Heep M, Rieger U, Beck D, Lehn N (2000). Mutations in the beginning of the rpoB gene can induce resistance to rifamycins in both Helicobacter pylori and Mycobacterium tuberculosis. Antimicrob Agents Chemother.

[24] Campbell EA, Korzheva N, Mustaev A (2001). Structural mechanism for rifampicin inhibition of bacterial RNA polymerase. Cell.

[25] Prasad R, Gupta N, Banka A (2018). Multidrug-resistant tuberculosis/rifampicin-resistant tuberculosis: principles of management. Lung India.

